# Identification of a key gene module associated with glucocorticoid- induced derangement in bone mineral density in patients with asthma

**DOI:** 10.1038/s41598-019-56656-9

**Published:** 2019-12-27

**Authors:** Suh-Young Lee, Ha-Kyeong Won, Byung-Keun Kim, Sae-Hoon Kim, Yoon-Seok Chang, Sang-Heon Cho, H. William Kelly, Kelan G. Tantisira, Heung-Woo Park

**Affiliations:** 1 Department of Internal Medicine, Seoul National University College of Medicine, Seoul, Republic of Korea; 2 Institute of Allergy and Clinical Immunology, Seoul National University Medical Research Center, Seoul, Republic of Korea; 3 Department of Internal Medicine, Korea University Medical Center Anam Hospital, Seoul, Republic of Korea; 4 Department of Internal Medicine, Seoul National University Bundang Hospital, Seongnam, Gyeonggi-do Republic of Korea; 5 Department of Pediatrics, University of New Mexico Health Sciences Center, Albuquerque, NM USA; 6 The Channing Division of Network Medicine, Department of Medicine, Brigham and Women’s Hospital, Harvard Medical School, Boston, MA USA; 7 Division of Pulmonary and Critical Care Medicine, Department of Medicine, Brigham and Women’s Hospital, Boston, MA USA

**Keywords:** Genetics, Asthma

## Abstract

Derangement in bone mineral density (BMD) caused by glucocorticoid is well-known. The present study aimed to find key biological pathways associated with low BMD after glucocorticoid treatment in asthmatics using gene expression profiles of peripheral blood cells. We utilized immortalized B cells (IBCs) from 32 childhood asthmatics after multiple oral glucocorticoid bursts and peripheral blood mononuclear cells (PBMCs) from 17 adult asthmatics after a long-term use of oral glucocorticoid. We searched co-expressed gene modules significantly related with the BMD Z score in childhood asthmatics and tested if these gene modules were preserved and significantly associated with the BMD Z score in adult asthmatics as well. We identified a gene module composed of 199 genes significantly associated with low BMD in both childhood and adult asthmatics. The structure of this module was preserved across gene expression profiles. We found that the cellular metabolic pathway was significantly enriched in this module. Among 18 hub genes in this module, we postulated that 2 genes, *CREBBP* and *EP300*, contributed to low BMD following a literature review. A novel biologic pathway identified in this study highlighted a gene module and several genes as playing possible roles in the pathogenesis of glucocorticoid- induced derangement in BMD.

## Introduction

Derangement in bone mineral density (BMD) caused by glucocorticoids is well-known. Glucocorticoid treatment is the most common cause of secondary osteoporosis^1^ and can induce a dosage-dependent reduction in bone mineral accretion in childhood asthmatics^2^. We previously reported that polymorphisms located in *CRHR1*, *TBCD* and *RAPGEF5* were associated with low BMD in childhood asthmatics after a long-term treatment of glucocorticoids^3–5^. However, so far, there has been no comprehensive study to elucidate biologic mechanisms underlying glucocorticoid-induced derangement in BMD in asthmatics who are frequently treated by glucocorticoid.

A network based analysis is an emerging and promising approach and provides new insights into BMD genetics by grouping genes into modules which are enriched for particular biological processes^6,7^. The present study aimed to find key biological pathways associated with low BMD after glucocorticoid treatment in patients with asthma using weighted gene co-expression network analysis (WGCNA).

For this purpose, we used gene expression profiles of immortalized B cells (IBCs) from childhood asthmatics after multiple oral glucocorticoid bursts and those of peripheral blood mononuclear cells (PBMCs) from adult asthmatics after a long-term use of oral glucocorticoids. Peripheral blood is an easily accessible tissue, although it is not directly involved in bone biology. However, it was suggested that molecular profiling of peripheral blood might reflect physiological and pathological events occurring in different tissues of the body^8^. Moreover, recent reports showed that PBMCs could be used to assess glucocorticoid sensitivity or efficacy of osteoporosis treatment^9,10^. Taken together, we may use gene expression profiles of peripheral blood cells for the purpose of searching what biologic pathways are related to glucocorticoid-induced derangement in BMD.

## Results

**Figure 1:**
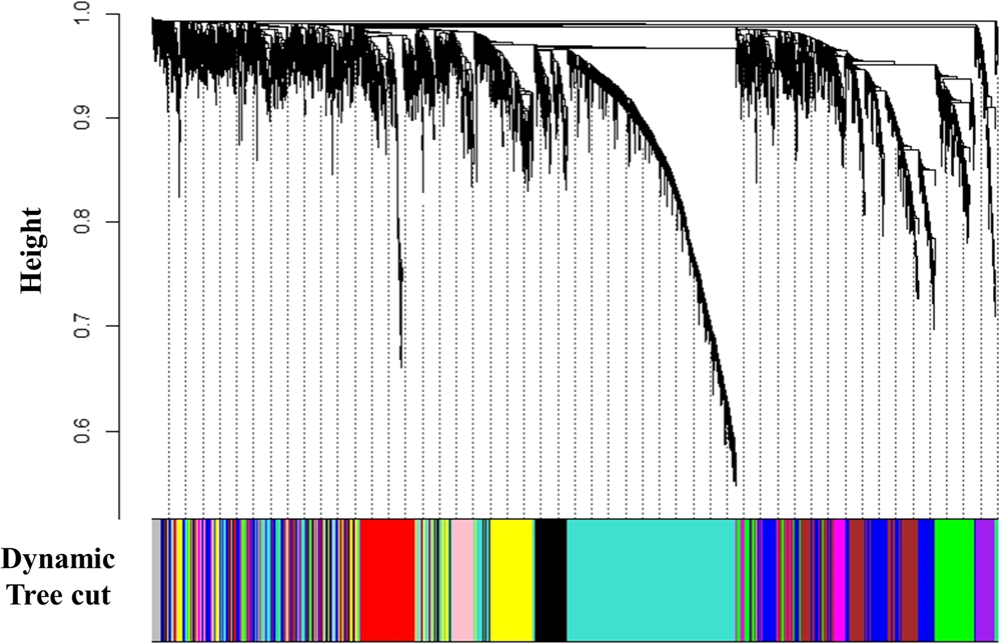
Co-expression modules identified in gene expression profiles of Sham-treated immortalized B cells from childhood asthmatics. Top panel shows a dendrogram of 5,000 genes. Bottom panel shows colors corresponding to the cluster membership labels for each gene.

**Figure 2:**
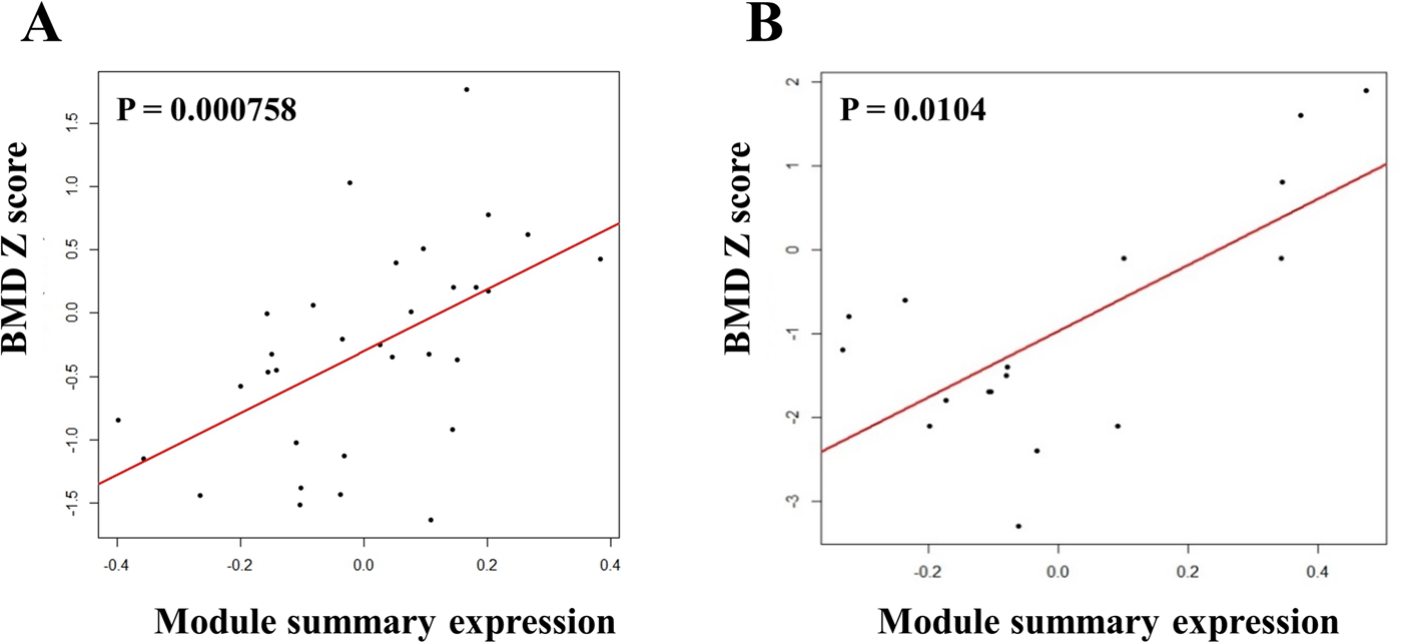
Associations between eigengene values of the bone mineral density Z score-associated common module and the bone mineral density Z score. (**A**) Childhood asthmatics. (**B**) Adult asthmatics. Both P values were adjusted by covariates.

A total of 32 childhood asthmatics whose IBCs and BMD profiles were available and 17 adult asthmatics enrolled (Table 1). Applying WGCNA to the 5,000 genes expressed in Sham-treated IBCs, we could identify 10 modules with various sizes ranging from 125 genes in the purple module to 1,372 genes in the turquoise module (Fig. 1). To emphasize the impact of strong correlations over weak ones in the network construction, we chose an empirical soft threshold of 6, representing a strong model fit for scale-free topology (R^2^ > 0.8, Figure S1). A total of 63 genes could not be assigned to a module as a membership gene, and were grouped into the grey module which was not considered for further analysis. Eigengene values of two modules (blue and magenta modules) showed significant associations with the BMD Z score in multivariate linear regression analysis. Eigengene value of the blue module consisted of 794 genes was negatively associated with the BMD Z score (P = 0.00294), whereas that of the magenta module containing 199 genes showed a positive association (P = 0.000758) (Fig. 2 and Figure S2).

**Table 1:** Characteristics of subjects enrolled.

	Childhood asthmatics^†^ (n = 32)	Adult asthmatics^‡^ (n = 17)
Gender, male (%)	23 (71.9)	6 (35.3)
Age, year	8.63 (1.93)	60.11 (11.71)
Smoking, yes (%)	Not applicable	3 (17.65)
Body mass index, kg/m^2^	17.71 (3.21)	23.72 (5.18)
Vitamin D, ng/mL	31.81 (9.97)	14.71 (7.72)
Tanner stage (%)		
I/II/III/IV/V	24 (75.0)/6 (18.8)/1(3.1)/1 (3.1)	Not applicable
Baseline BMD Z-score	−0.10 (2.23)	Not available
Ethnicity (%)		
Non-Hispanic white	32 (100)	0
Asian	0	17 (100)
Cumulative dose of PD, mg	1188.40 (608.05)	3130.53 (1552.09)
Final BMD Z-score	−0.29 (0.81)	−0.97 (1.37)

Data indicate mean (Standard deviation); BMD, Bone mineral density; PD, Prednisone; ^†^Characteristics measured at enrollment of the CAMP study except cumulative dose of prednisone and the final BMD Z-score; ^‡^Characteristics measured at enrollment of the present study.

**Figure 3:**
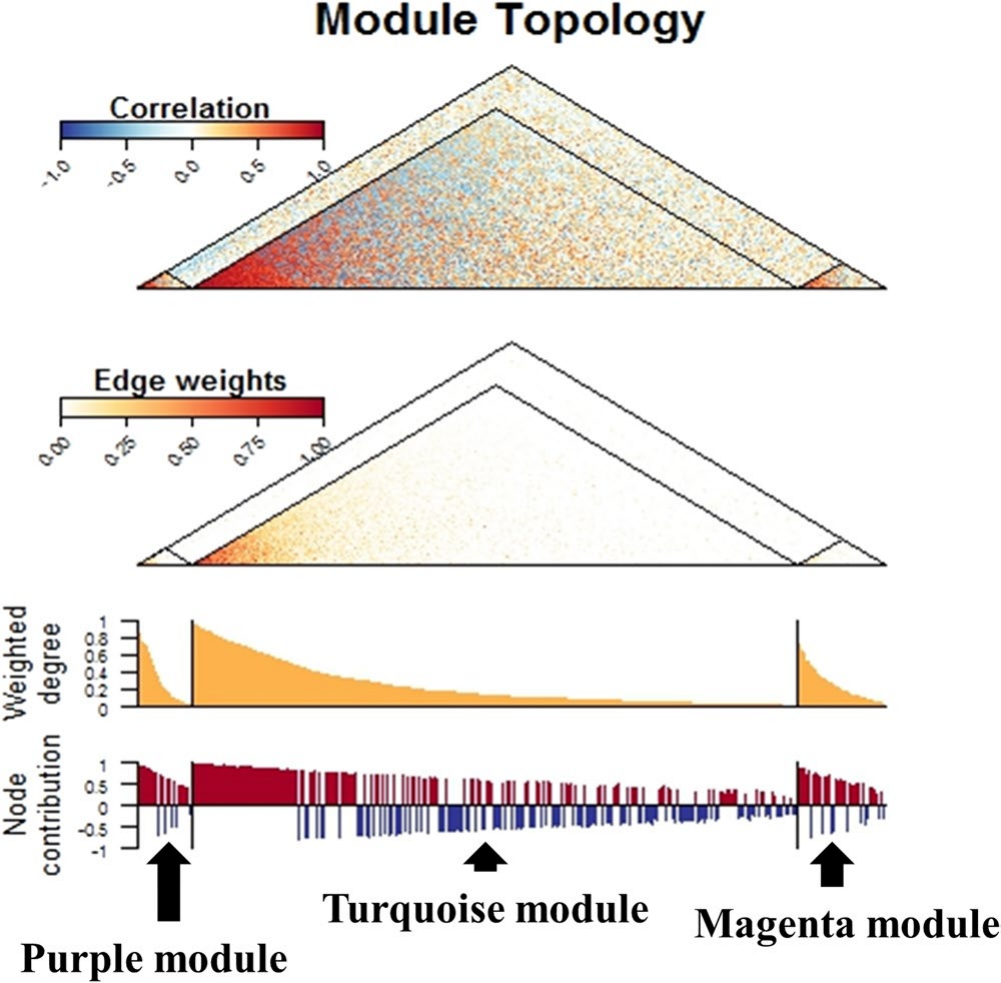
Preservation of modules identified in gene expression profiles of Sham-treated immortalized B cells from childhood asthmatics in gene expression profiles of Sham-treated peripheral mononuclear cell from adult asthmatics. The first (top) panel shows a heatmap of pair-wise correlations among the genes comprising the turquoise, magenta, and purple modules. The second panel shows a heatmap of the edge weights (connections) among the genes comprising the three modules. The third panel shows the distribution of scaled weight degrees (relative connectedness) among the genes comprising the three modules. The fourth panel shows the distribution of node contributions (correlation to module eigengene) among the genes comprising the three modules. Genes are ordered from left to right based on their weighted degree in the discovery cohort, so as to highlight the consistency of the network properties in the replication cohort.

To assess the reproducibility and generalizability, we tested if our results were replicated in other gene expression profiles from adult asthmatics (Sham-treated PBMCs). Replication analysis was done in two ways: preservation of module (the consistency of network module structure across gene expression profiles) and association with the target phenotype (association between corresponding eigengene values and the BMD Z score). Among 10 modules identified in the discovery cohort, three modules (turquoise, magenta and purple modules) were significantly preserved in the replication cohort (Fig. 3). Module preservation statistics and P values are presented in Table S1. Multivariate linear regression analysis showed that eigengene value of the magenta module was significantly associated with the BMD Z score in the replication cohort (P = 0.0104) (Fig. 2). Based on these findings, we defined the magenta module identified in gene expression profiles from childhood asthmatics as the BMD Z score-associated common module. Among 199 membership genes in the BMD Z score-associated common module, 18 genes (*ARMC5, ATP2A2, CCNK, CREBBP, EP300, EP400, GTF3C1, IPO13, MTF1, NOL8, NUP188, PCF11, RFX5, SDAD1, SETD1A, SLC25A22, UBAP2L, WDR59*) were taken as hub genes due to their high connectivity and association with the BMD Z score (Figure S3 and Table S2).

**Figure 4:**
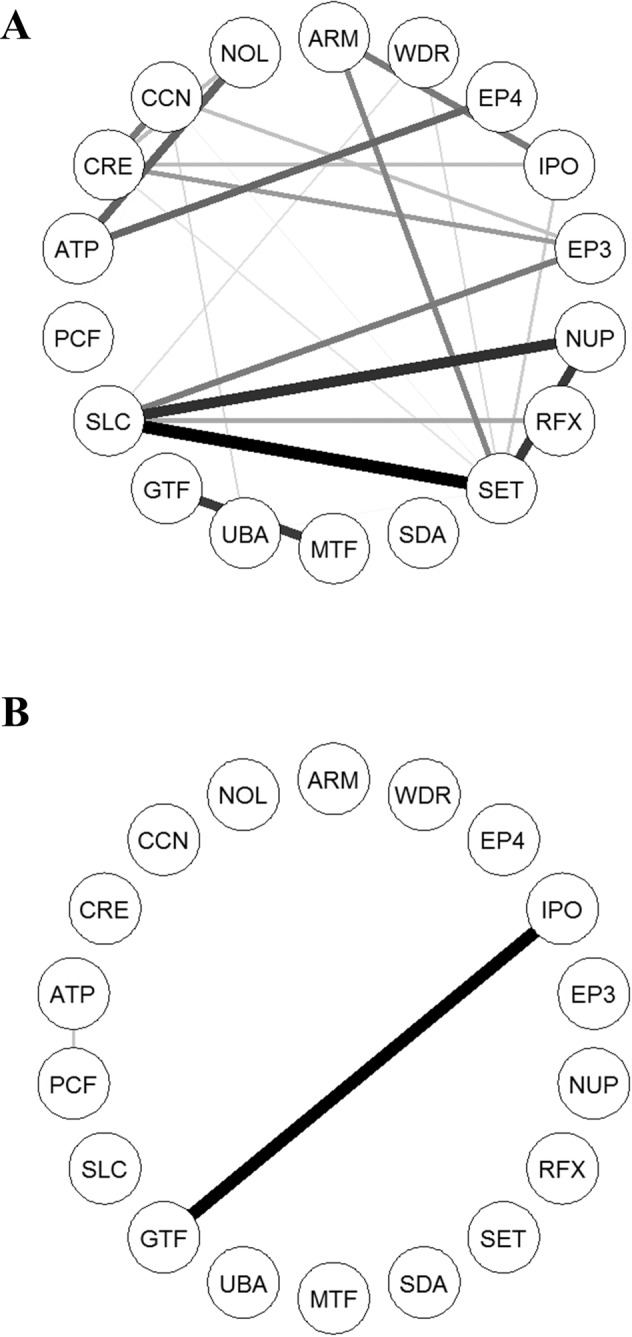
Co-expression network of hub genes of the bone mineral density Z score-associated common module. (**A**) Sham-treated IBCs from childhood asthmatics. (**B**) Dex-treated IBCs from childhood asthmatics. For clarity, only the edges corresponding to the Pearson correlation coefficient >0.8 were shown. The edge width is proportional to the Pearson correlation coefficient between two nodes. The network was visualized using qgraph R package. ARM*, ARMC5;* ATP*, ATP2A2;* CCN*, CCNK;* CRE*, CREBBP;* EP3*, EP300;* EP4*, EP400;* GTF*, GTF3C1;* IPO*, IPO13;* MTF*, MTF1;* NOL*, NOL8;* NUP*, NUP188;* PCF*, PCF11;* RFX*, RFX5;* SDA*, SDAD1;* SET*, SETD1A;* SLC*, SLC25A22;* UBA*, UBAP2L;* WDR*, WDR59*.

To evaluate *in vitro* perturbation of modules identified in gene expressions of Sham-treated IBCs by Dex, we measured changes of the module structure between Sham- and Dex-treated IBCs. Among the 10 modules, only the magenta module identified in Sham-treated IBCs was not preserved in Dex-treated IBCs (a maximal permutation P = 0.096, Table S3). In addition, a co-expression network of the 18 hub genes showed distinct differences between them (Fig. 4).

To assign biological meaning to interpretability of the BMD Z score-associated common module, we performed pathway enrichment analyses in GO biological process categories with its membership genes. To increase the specificity in the enrichment results, we set a more stringent threshold of overlap (intersection of query and test sets ≥ 3) and excluded consideration of inferred electronic annotations (i.e., annotations created by *in silico* curation methods of potentially lower quality). The most significantly enriched pathway was the cellular metabolic pathway (GO:0044237, FDR adjusted P value = 2.75E-7). This pathway consists of 10,751 genes and 150 genes out of 199 genes belong to the BMD Z score-associated common module overlapped. Enriched pathways identified are shown in Table 2. As we could not find datasets to replicate our findings, we performed a pathway analysis using the top-200 single nucleotide polymorphisms (SNPs) (Table S4) that were significantly associated with the bone mineral accretion in childhood asthmatics treated by oral prednisone bursts in our previous genome-wide association study^4^. We performed a pathway analysis using genes mapped to 200 SNPs and identify the cellular macromolecular metabolic process (GO:0044260) as the most significant candidate causal pathway (FDR P value = 0.02).

**Table 2:** Biological pathways enriched in the bone mineral density Z score-associated common module.

ID	Name^†^	Depth^‡^	P value^§^
GO:1903901	Negative regulation of viral life cycle	1	0.0418
GO:0045071	Negative regulation of viral genome replication	1	0.014
GO:0006475	Internal protein amino acid acetylation	1	0.0448
GO:0018394	Peptidyl-lysine acetylation	1	0.0453
GO:0018393	Internal peptidyl-lysine acetylation	2	0.0394
GO:0018076	N-terminal peptidyl-lysine acetylation	2	0.0287
GO:0016570	Histone modification	1	0.0291
GO:0016573	Histone acetylation	2	0.0352
GO:0010646	Regulation of cell communication	1	0.0485
GO:0051169	Nuclear transport	1	0.041
GO:0006913	Nucleocytoplasmic transport	2	0.0374
GO:0002886	Regulation of myeloid leukocyte mediated immunity	1	0.0321
GO:0002279	Mast cell activation involved in immune response	1	0.0453
GO:0051640	Organelle localization	1	0.0335
GO:0043300	Regulation of leukocyte degranulation	1	0.0336
GO:0051656	Establishment of organelle localization	1	0.0052
GO:0033003	Regulation of mast cell activation	1	0.0283
GO:0033006	Regulation of mast cell activation involved in immune response	2	0.0153
GO:0043303	Mast cell degranulation	1	0.0453
GO:0043304	Regulation of mast cell degranulation	2	0.0153
GO:0050657	Nucleic acid transport	1	0.0335
GO:0051236	Establishment of RNA localization	1	0.0354
GO:0050658	RNA transport	2	0.0335
GO:0006405	RNA export from nucleus	3	0.0287
GO:0071166	Ribonucleoprotein complex localization	1	0.0219
GO:0071426	Ribonucleoprotein complex export from nucleus	2	0.0205
GO:0008150	Biological process	1	0.00234
GO:0071840	Cellular component organization or biogenesis	2	0.0279
GO:0044085	Cellular component biogenesis	3	0.00104
GO:0008152	Metabolic process	2	0.00000288
GO:0044238	Primary metabolic process	3	0.000013
GO:0071704	Organic substance metabolic process	3	0.0000188
GO:0009058	Biosynthetic process	3	0.000516
GO:0006807	Nitrogen compound metabolic process	3	0.0000273
GO:0009987	Cellular process	2	0.000425
GO:0044237	Cellular metabolic process	3	0.000000275
GO:0043933	Macromolecular complex subunit organization	1	0.0278
GO:0071822	Protein complex subunit organization	2	0.019
GO:0065003	Macromolecular complex assembly	1	0.0289
GO:0006461	Protein complex assembly	2	0.0278
GO:0010629	Negative regulation of gene expression	1	0.0138
GO:0000122	Negative regulation of transcription from RNA polymerase II promoter	1	0.0272
GO:0002520	Immune system development	1	0.0138
GO:0048534	Hematopoietic or lymphoid organ development	2	0.0167
GO:0030097	Hemopoiesis	3	0.0225
GO:0006325	Chromatin organization	1	0.034
GO:0031648	Protein destabilization	1	0.0356
GO:0033554	Cellular response to stress	1	0.00328
GO:0036294	Cellular response to decreased oxygen levels	1	0.0448
GO:0071456	Cellular response to hypoxia	2	0.038
GO:0043618	Regulation of transcription from RNA polymerase II promoter in response to stress	1	0.0428
GO:0061418	Regulation of transcription from RNA polymerase II promoter in response to hypoxia	2	0.0466
GO:0051649	Establishment of localization in cell	1	0.0302

^†^Gene ontology biological pathway, ^‡^Only depth 1~3 were presented, ^§^Benjamini-Hocherg FDR P value.

## Discussion

The purpose of this study was to find key biological pathways associated with glucocorticoid-induced BMD derangement including a reduction in bone mineral accretion in childhood asthmatics and a reduction in BMD in adult asthmatics after long-term treatment of glucocorticoid. Using gene expression profiles on blood cells, we could identify a key gene module composed of 199 genes whose eigengene value showed a significant association with the BMD Z score in both childhood and adult asthmatics and whose connectivity was preserved as well.

Membership genes in this module were significantly overrepresented in the cellular metabolic pathway (GO:0044237). A previous report exploring differential gene expression profiles on PBMC by the graph clustering approach and GO term enrichment analysis showed 9 gene clusters associated with osteoporosis^11^. Nine clusters included various GO pathways, such as, response to virus (GO:0009615), immune response (GO:0006955), response to hypoxia (GO:0001666), and response to extracellular stimulus (GO:0009991) which were similar with GO pathways identified in this study. The cellular metabolic pathway representing the chemical reactions and pathways by which individual cells transform chemical substances never been recognized in previous studies focused on osteoporosis. To get a further insight, we performed a pathway analysis using the genes mapped to top-200 SNPs that were significantly associated with the bone mineral accretion in childhood asthmatics in our previous genome-wide association study^4^. We identified the cellular macromolecular metabolic process (GO:0044260) as the most important candidate causal pathway. This pathway is a sub-pathway of the cellular metabolic pathway (GO:0044237)^12^. These SNPs could not be directly linked to gene expression/modules identified in the present study, as genomics data were generated from different populations. However, we thought that this result would be an additional evidence suggesting a possible role of the cellular metabolic pathway in osteoporosis pathogenesis. Cellular metabolism is an important part of the bone biology and homeostasis^13,14^, but, so far, no direct evidence connecting this pathway to glucocorticoid-induced BMD derangement has been issued, although the cellular amine metabolic pathway (GO:0033240), a sub-pathway of the cellular metabolic pathway, was reported to be enriched in gene expressions of PBMC from patients with osteoporosis compared to normal control^15^. Further studies are needed to confirm our observations.

It was not easy to give biological meanings to our findings at the pathway level, as identified pathways were diverse and contained many genes (for example, the cellular metabolic pathway included 9416 genes^12^). For this reason, we selected highly connected intramodular18 hub genes. By reviewing the literatures, we selected two genes of putative interest: *EP300* encoding E1A binding protein p300 and *CREBBP* coding CREB binding protein (CBP). CBP and p300 are highly related transcriptional co‐activators possessing histone acetyltransferase activity and are known to participate in the activities of hundreds of different transcription factors involved in a variety of cell functions^16^. Up-regulation of miR-132-3p could inhibit osteoblast differentiation in part by decreasing p300 expression^17^, and CBP could mediate osteoblast-adipocyte lineage plasticity by interaction with Maf^18,19^. In addition, both CBP and p300 were known to be important co-factors mediating transforming growth factor-beta and bone morphogenic protein signaling which have fundamental roles in bone homeostasis^20^, and to modulate parathyroid hormone regulation of osteoblast^21^. Interestingly, it has been also reported that these two proteins are involved in steroid receptor signaling^22^. Steroid receptors require coactivators for efficient activation of target gene expression and these coactivators might contribute to tissue specific responses to steroid hormone. Likewise, CBP and p300 might function additively or antagonistically to each other depending on their relative concentrations and type of target tissue, to influence the sensitivity of tissues to glucocorticoids^23^. In addition, it was reported that *CREBBP* mutations resulted in impaired expression of GC receptor responsive gene and impaired histone acetylation and transcriptional regulation^24,25^. Given that CBP and p300 are involved not only in bone homeostasis but also in glucocorticoid receptor signaling, a gene module identified in this study which contained *CREBBP* and *EP300* may play an important role in glucocorticoid-induced derangement in BMD.

Among other genes belong to the hub genes, *ARMC5* encoding armadillo repeat containing 5 and *IPO13* encoding importin 13 are of interest. *ARMC5* inactivation affects steroid production and cell survival *in vitro* and adrenal hyperplasia associated with severe Cushing syndrome^26^. As well known, Cushing’s syndrome results in osteoporosis, which is analogue to glucocorticoid-induced osteoporosis^27^. *IPO13* is a steroid-inducible gene and the glucocorticoid receptor is a cargo substrate for importin 13^28^. IPO13 is thus possibly associated with glucocorticoid signaling, although its role in glucocorticoid-induced osteoporosis is unknown.

The strength of our study was that we searched biological mechanisms underlying glucocorticoid-induced derangement in BMD with a network based methodology for the first time. A recent review highlighted an importance of network-based approach in genomic studies for osteoporosis^29^. Considering that the modularity of networks is inherent to cell biology and thus molecular interactions organized into functional unit are more relevant to explain biological phenomena^30^, the present study might provide a comprehensive and holistic understanding of glucocorticoid-induced derangement in BMD. To generate biological networks, we utilized WGCNA in the present study. WGCNA, unlike the other biological networks, is a way to organize data in an unbiased manner and provides a gene module summary which can be related with clinical phenotypes^29^. In a systems genetics aspect, if a Bayesian structure learning algorithm is to be applied to WGNCA, the direction of the flow of molecular information would be elucidated, which enables us figure out the causal relationship. Recently, such an attempt identified novel pathways in patients with the late-onset Alzheimer’s disease pathology and coronary artery disease^31,32^. However, to our knowledge, Bayesian structure learning algorithm in WGNCA has not yet been applied in the field of bone mineral density and steroid response. Another important thing is that we can analyze co-expressions across a set of perturbations with WGCNA. In the present study, we found that a structure of the BMD Z score-associated common module was disrupted by *in vitro* perturbation using gene expression profiles of Dex-treated IBCs. Eigengene values of this module showed significant positive associations with the BMD Z score in both childhood and adult asthmatic (Fig. 2). We assume that a disruption of this protective gene module by glucocorticoid may result in BMD reduction in asthmatics treated by glucocorticoid for a long time, which provides a functional relevance of our observations. In addition, we confirmed that a module significantly associated with the BMD Z score preserved in different conditions (bone mineral accretion in childhood asthmatics and osteoporosis in adult asthmatics), which may increase the generalizability of observations in this study.

There are some limitations in our study. First, we utilized gene expression profiles of blood cells instead of the cells directly involved in bone biology. We could find a publicly available gene expression dataset of Sham- and Dex-treated osteoblast (GSE21727)^33^. This dataset was generated from cultured trabecular bone cells obtained from ~100 unrelated Caucasian donors treated by Sham and Dex (100 nM, 2 hour later). Using this dataset, we could find that the magenta module (the BMD Z score-associated common module) identified in this study was preserved in gene expressions of Sham-treated osteoblasts (a maximal permutation P = 0.0000599; Table S5 and Figure S4). Similar to Dex-treated IBCs, this module was not preserved in gene expressions of Dex-treated osteoblasts (a maximal permutation P = 0.253; Data were not shown). These findings suggested that a gene modules of this study might be important across different tissue types (blood cells and osteoblast), although GSE21727 was a small dataset (one subject with three replicates). Evidently, previous reports showed that tissue-specific genes can be expressed in non-tissue-specific manner^34,35^, and peripheral blood cells expressed approximately over 80% of the genes encoded by the human genome^36^. In addition, it was demonstrated that osteoclasts could be generated from PBMC population^37,38^ and PBMC could be used to assess efficacy of osteoporosis treatment^10^. Taken together, peripheral blood cells may be a surrogate for bone tissue and recapitulate bone cell biology in part, although further studies will be needed. Secondly, a small number of participants, different ethnicity and age (Non-Hispanic white childhood asthmatics *vs*. Asian elderly asthmatics), and different ways of exposure to glucocorticoid (short-term burst *vs*. chronic use) were considered before generalizing our findings. Previously, age-dependent alterations in osteoblast and osteoclast activity were reported^39,40^. In general, short-term glucocorticoid use was associated with mild adverse effects, whereas long-term use might be associated with more serious sequel including osteoporosis^41^. For this reason, we provided information on the hub genes and enriched biologic pathways of genes belong to the blue module, a childhood asthmatics-specific gene module, in the supplementary information (Figure S5, Table S6, and Table S7). Currently, there are no publicly available datasets to validate our findings. However, a replication analysis should be done when datasets become available. In addition, an integrative analysis using other potentially relevant -omics data such as, miRNAs, lncRNAs, and methylation profiles is warranted in the future.

In conclusion, the potentially significant genes and pathways identified in our analysis may help further elucidate of the molecular mechanisms and provide novel insights regarding glucocorticoid-induced derangement in BMD in asthmatics.

## Methods

**Figure 5:**
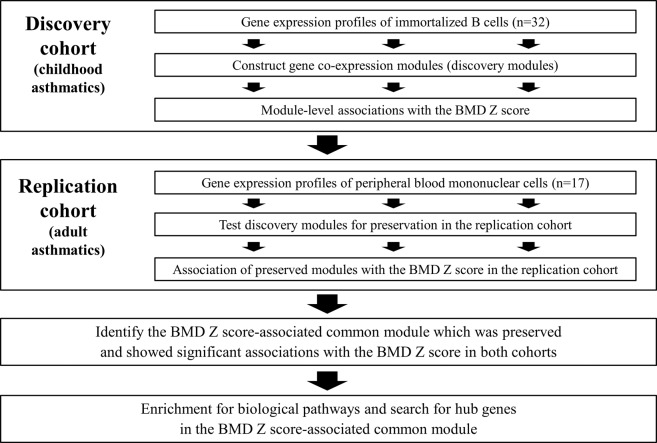
Overall study design.

All experimental protocols were approved by the Institutional Review Board of the corresponding institutions. This study was approved by the Institutional Review Board of the Brigham and Women’s Hospital (2002-P-00331/41) and the Seoul National University Hospital (H-1508-095-695). Informed consents were obtained from all study participants and a parent and/or legal guardian, if subjects were under 18. And all methods were carried out in accordance with relevant guidelines and regulations. The overall study design is presented in Fig. 5.

### Study populations and bone mineral density measurements

The discovery cohort consisted of Non-Hispanic white children from the Childhood asthma management program (CAMP) trial whose IBCs were available^42^. In asthma exacerbations, short courses of oral prednisone were prescribed per protocol to them. Each burst consisted of 2 mg/kg per day (up to 60 mg) of prednisone for 2 days followed by 1 mg/kg per day (up to 30 mg) for 2 days. Subsequent maintenance of prednisone was allowed when the improvement was not sufficient. BMD measurements of the lumbar spine (L1-L4) were performed annualy during the study period by means of dual-energy x-ray absorptiometry [Hologic (Hologic Inc., Waltham, MA, US) or Lunar (GE Healthcare, Madison, WI, US)]. The BMD Z score that represents a difference between the measured bone density in a subject and the average bone density in age- and sex-matched controls was calculated by using the CAMP internal ref. ^2^. In the present study, the final BMD Z score measured after mean follow-up duration of 4.3 years was used as a target phenotype.

The replication cohort was drawn from adult asthmatics who were treated at Seoul National University Hospital, Seoul, Korea. All of them received more than 15 mg of prednisone per day continuously for at least one year before enrollment for the control of their symptoms. Doses above 10 mg of prednisone per day result in significant bone loss^43^. At the enrollment, BMD was measured at the lumbar spine (L1-L4) by dual-energy X-ray absorptiometry [Lunar (GE healthcare, Madison, WI, US)]. Like childhood asthmatics, the BMD Z score was used as a target phenotype.

### Gene expression arrays

As previously described^44^ IBCs derived from childhood asthmatics from the CAMP trial were cultured in RPMI 1640 medium (Sham-treated IBCs) and treated with dexamethasone (10^−6^ M) for 6 hours (Dex-treated IBCs). Dex-treated IBCs were used to measure *in vitro* perturbation of gene expressions by Dex. PBMCs from adult asthmatics were cultured in RPMI 1640 medium for 6 hours (Sham-treated PBMCs). Gene expression levels were measured using the Illumina HumanRef8 v2 BeadChip (Illumina, San Diego, CA, US) for childhood asthmatics and the Affymetrix GeneChip Human Gene 2.0 ST (Affymetrix, Santa Clara, CA, US) for adult asthmatics. We removed probes with bad chromosome annotation and probes in X or Y chromosome. We then did variance stabilizing transformation and quantile normalization respectively to reduce the effects of technical noises and to make the distribution of expression level for each array closer to normal distribution.

### Statistical analysis

Analysis was performed with R version 3.4.3 (www.r-project.org). We performed WGCNA on each gene expression profile with the R package “WGCNA”^45^. To allow comparability of data from different microarray platforms, we first calculated the mean expression value of each gene in two expression profiles (Sham-treated IBCs and Sham-treated PBMCs) after collapsing probes by genes. We then compared the mean expression level of genes of them and selected top-5000 genes in common with the highest correlation. Using 5,000 genes we first characterized unsigned correlation networks and their relationships with each other and found gene modules in gene expression profiles from childhood asthmatics. A module was defined as a group of highly interconnected genes^45^. We computed eigengene values of modules identified and performed multivariate linear regression analysis adjusted by baseline age, gender, BMD Z score, vitamin D level, Tanner stage, and body mass index Z score. Eigengene values can be effective and biologically meaningful tools for studying the relationships between modules of a gene co-expression network and phenotypes^46^. The BMD Z score-associated module was defined when its eigengene value was significantly associated with the BMD Z score, a target phenotype. We next tested if the BMD Z score-associated modules identified from childhood asthmatics and were preserved in gene expression profiles from adult asthmatics using the R package “NetRep”^47^. NetRep can quantify the preservation of gene co-expression modules across different datasets and produce unbiased p values based on a permutation approach to score module preservation without assuming data are normally distributed^47^. The BMD Z score-associated common module denoted a module which was identified in gene expression profiles from childhood asthmatics and significantly preserved in gene expression profiles from adult asthmatics. We then calculated an eigengene value (the first principle component) of the BMD Z score-associated common module in adult asthmatics and performed multivariate linear regression analysis adjusted by age, gender, vitamin D level, body mass index, and smoking status.

We then performed Gene ontology (GO) pathway overrepresentation analyses of the BMD Z score-associated common modules using “g:Profiler” (database version: r1730_e88_eg35)^48^. g:Profiler (https://biit.cs.ut.ee/gprofiler/) provides an adjusted P value calculated in a manner that accounts for the hierarchical relationships among the tested gene sets. Finally, we searched the key driving hub genes in the BMD Z score-associated common modules, as an occurrence of certain disease is less likely associated with the abnormality of the whole genes involved in the biological pathway. Hub genes were defined by module connectivity, measured by absolute value of the Pearson’s correlation (cor.geneModuleMembership > 0.8) and clinical trait relationship, measured by absolute value of the Pearson’s correlation (cor.geneTraitSignificance >0.2)^45,49^. For the pathway analysis of SNPs, we selected the top-200 SNPs (Table S) from our previous report^4^ and put those SNPs into ICSNPathway (http://icsnpathway.psych.ac.cn/). The ICSNPathway enables us to identify candidate causal SNPs and pathways from genome-wide association study by one analytical framework^50^. Parameters used were as follow: specify SNP, P value < 1.0E-5; linkage disequilibrium cutoff, *r*^2^ = 0.8; Rule of mapping SNPs to genes, within gene; Population, CEU; Pathway gene set size, minimum = 5 and maximum = 100; FDR cutoff, 0.05.

## Supplementary information

Supplementary Information.

## Data Availability

Due to the regulation of the Ethics Review Board of our institute, we could not deposit our data in publicly available repositories. However, immediately following publication, individual participant data that underlie the results reported in this article will be able to be shared after de-identification with researchers who will provide a methodologically sound proposal. Proposals should be directed to guinea71@snu.ac.kr.
