# Supplementary material for: Identification of a key gene module associated with glucocorticoid- induced derangement in bone mineral density in patients with asthma
Source: Sci Rep. 2019 Dec 27;9:20133. doi: 10.1038/s41598-019-56656-9 (PMC6934743; doi:10.1038/s41598-019-56656-9)
Supplement: Supplementary file 1 — Supplementary Information. [file 41598_2019_56656_MOESM1_ESM.pdf]

Supplementary information

Identification of a key gene module associated with glucocorticoid induced derangement in bone mineral density in patients with asthma

Suh-Young Lee, MD,[1] Ha-Kyeong Won, MD,[1] Byung-Keun Kim, MD,[3] Sae-Hoon Kim, MD, PhD,[4] Yoon-Seok Chang, MD, PhD,[4] Sang-Heon Cho, MD, PhD,[1,2] H. William Kelly, PharmD,[5] Kelan G. Tantisira, MD, MPH,[6,7] Heung-Woo Park, MD, PhD[1,2,6]

[1]Department of Internal Medicine, Seoul National University College of Medicine, Seoul, Republic of Korea

[2]Institute of Allergy and Clinical Immunology, Seoul National University Medical Research Center, Seoul, Republic of Korea

[3]Department of Internal Medicine, Korea University Medical Center Anam Hospital, Seoul, Republic of Korea

[4]Department of Internal Medicine, Seoul National University Bundang Hospital, Seongnam, Gyeonggi-do, Republic of Korea

[5]Department of Pediatrics, University of New Mexico Health Sciences Center, Albuquerque, NM, USA

[6]The Channing Division of Network Medicine, Department of Medicine, Brigham and Women's Hospital, Harvard Medical School, Boston, MA, USA

[7]Division of Pulmonary and Critical Care Medicine, Department of Medicine, Brigham and Women's Hospital, Boston, MA, USA

Figure S1. Assessing scale-free model fitting in gene expression profiles Sham-treated immortalized B cells from childhood asthmatics

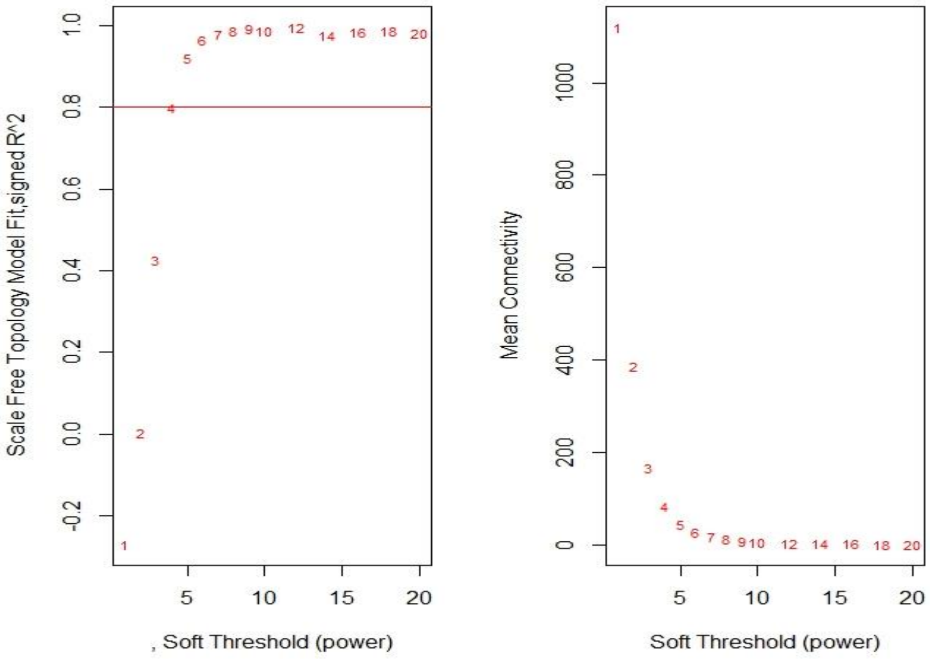

Left panel shows scale-free topology plotted by soft threshold. The red horizontal line represents the cutoff for identifying a strong model fit. Right panel shows mean gene connectivity plotted by soft threshold.

Figure S2. Correlations between eigengene values of two modules identified in gene expression profiles of Sham-treated immortalized B cells from childhood asthmatics and the bone mineral density Z score

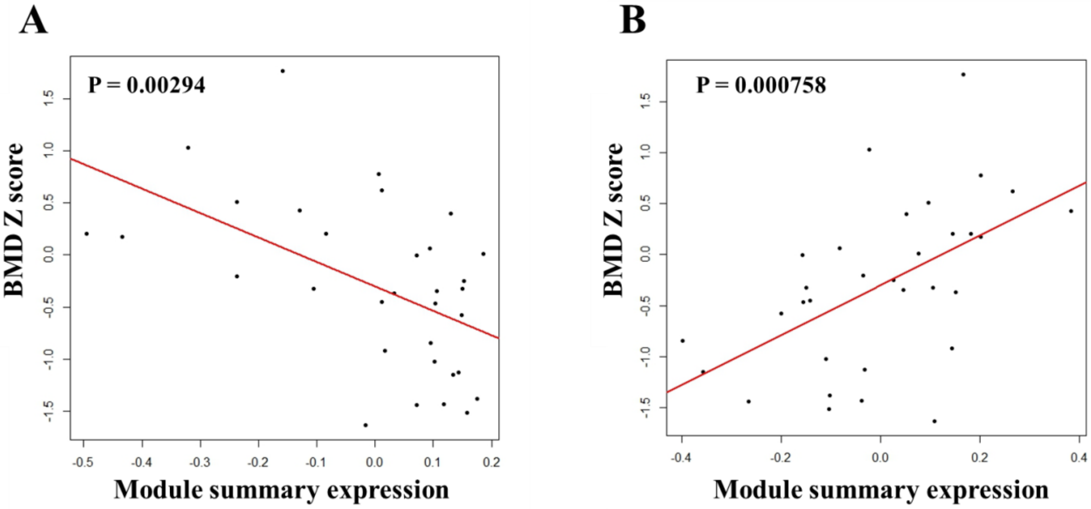

A, Blue gene module; B, Magenta gene module

All P values were adjusted by covariates.

Figure S3. Scatter plot of module eigengenes in the magenta module (bone mineral density Z score-associated common module)

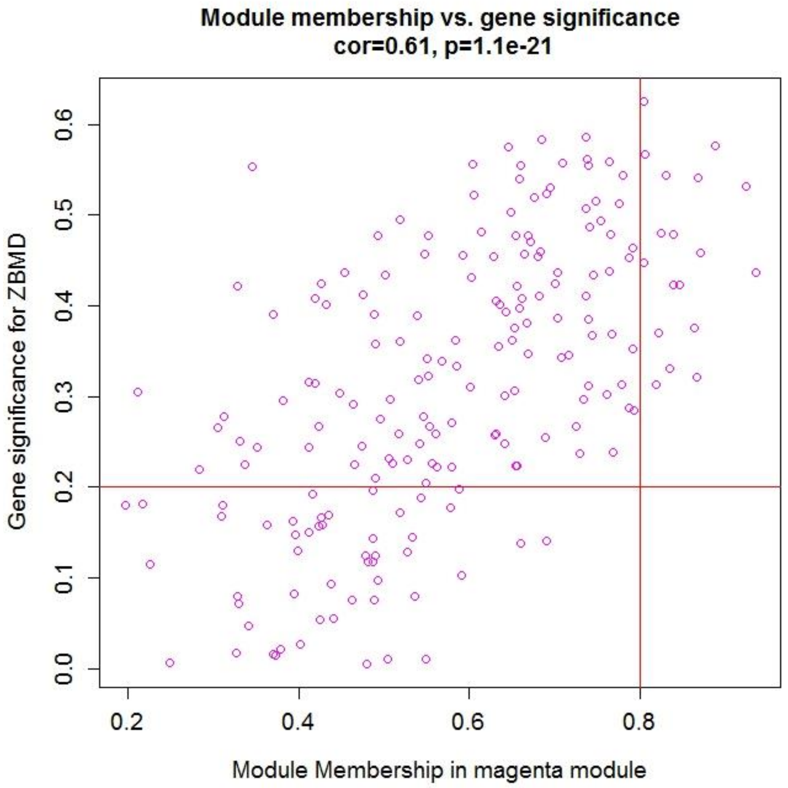

Hub genes are located at the right and upper quadrant.

Figure S4. Preservation of modules identified in gene expression profiles of Sham-treated immortalized B cells from childhood asthmatics in gene expression profiles of Sham-treated osteoblast from GSE21727

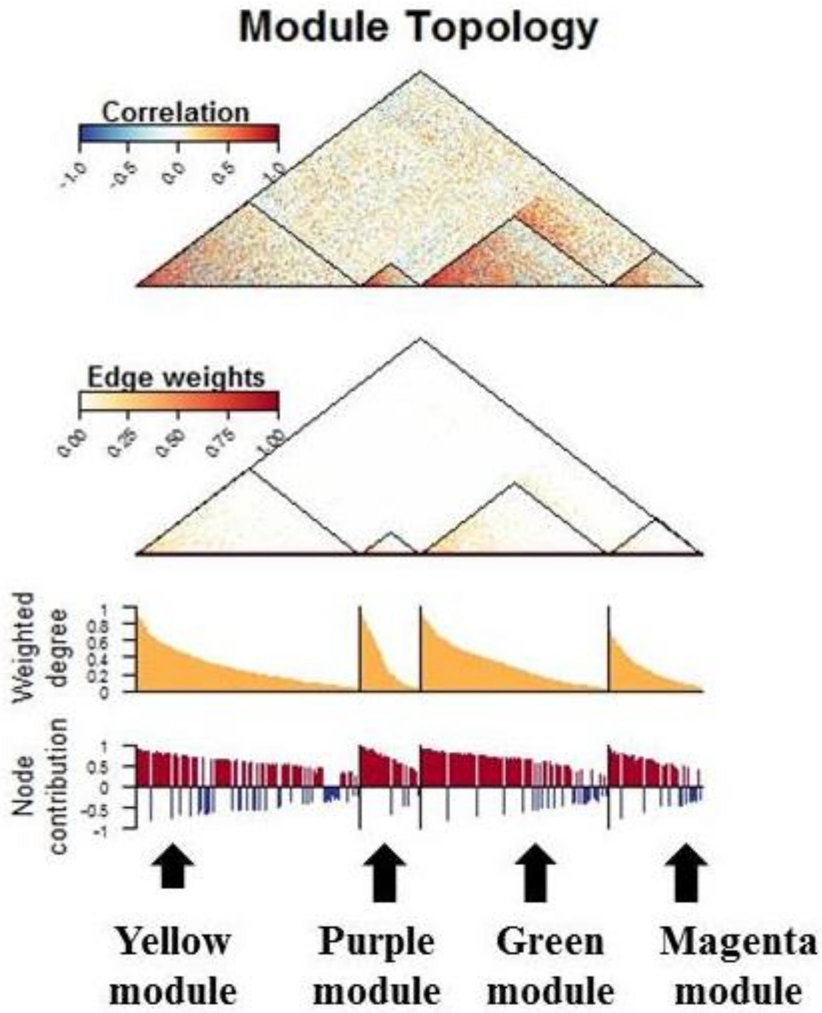

Figure S5. Scatter plot of module eigengenes in the blue module (childhood asthmatics-specific gene module)

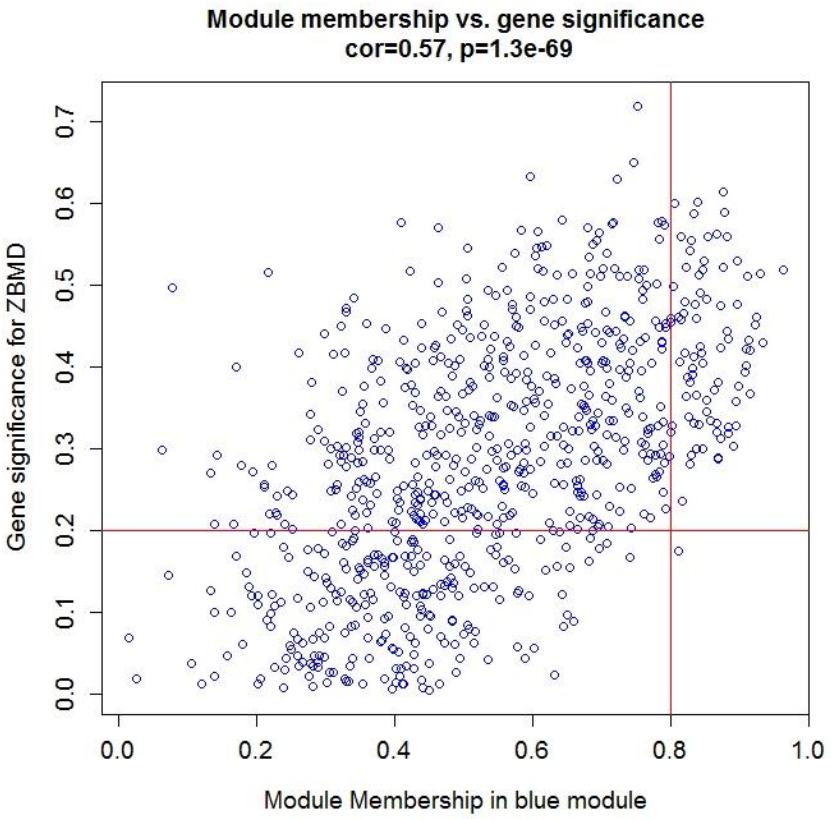

Hub genes are located at the right and upper quadrant.

Table S1. Preservation statistics and P values of modules identified in gene expression profiles of Sham-treated immortalized B cells from childhood asthmatics in gene expression profiles of Sham-treated peripheral mononuclear cell from adult asthmatics

	Module	avg.weight	coherence	cor.cor	cor.degree	cor.contrib	avg.cor	avg.contrib
**Statistics**	Turquoise	0.3189273	0.2815515	0.10167908	0.232922271	0.302193872	0.023312781	0.130860688
	Magenta	0.3019563	0.2635123	0.17010856	0.184381214	0.372697861	0.09403571	0.316896154
	Purple	0.2611963	0.2256738	-0.02868263	0.021443337	-0.188416992	0.008446526	0.055997275
**P values**	Turquoise	0.00009999	0.00049995	0.00009999	0.00009999	0.00009999	0.00009999	0.00009999
	Magenta	0.00009999	0.00009999	0.00009999	0.0059994	0.00009999	0.00009999	0.00009999
	Purple	0.00219978	0.00009999	0.00009999	0.00009999	0.00009999	0.00009999	0.00009999

Columns correspond to seven module preservation statistics defined by the “NetRep” package and P values are permutation P values.

'avg.weight' measures the average magnitude of edge weights in the test dataset, that is, how connected nodes in the module are to each other on average.

'coherence' measures the proportion of variance in the module data explained by the module's summary profile vector in the test dataset.

'cor.cor' measures the concordance of the correlation structure, that is, how similar the correlation heatmaps are between the two datasets.

'cor.degree' measures the concordance of the weighted degree of nodes between the two datasets, that is, whether the nodes that are most strongly connected in the discovery dataset remain the most strongly connected in the test dataset.

'cor.contrib' measures the concordance of the node contribution between the two datasets. This measures whether the module's summary profile summarizes the data in the same way in both datasets.

'avg.cor' measures the average magnitude of the correlation coefficients of the module in the test dataset, that is, how tightly correlated the module is on average in the test dataset. This score is penalized where the correlation coefficients change in sign between the two datasets.

'avg.contrib' measures the average magnitude of the node contribution in the test dataset. This is a measure of how coherent the data is in the test dataset. This score is penalized where the node contribution changes in sign between the two datasets, for example, where a gene is differentially expressed between the two datasets.

Table S2. Membership genes of the bone mineral density Z score-associated common module

Gene	Dexamethasone-responsiveness[†]		Cellular metabolic process gene[‡]
	log2 fold change	P value	
**ARMC5**	-0.0380249	0.5828633	No
**ATP2A2**	0.4167439	0.004790925	No
**CCNK**	-0.03142452	0.8246246	Yes
**CREBBP**	0.3673004	0.01933958	Yes
**EP300**	0.1895247	0.04375724	Yes
**EP400**	0.08339024	0.4198441	Yes
**GTF3C1**	-0.09396902	0.3400058	Yes
**IPO13**	-0.216802	0.03394458	Yes
**MTF1**	0.09902032	0.2934905	Yes
**NOL8**	0.4393377	0.004976667	Yes
**NUP188**	0.2586276	0.03014498	Yes
**PCF11**	0.363114	0.01749985	Yes
**RFX5**	-0.7230053	4.46E-05	Yes
**SDAD1**	-0.000207901	0.9989959	No
**SETD1A**	0.05012735	0.6606172	Yes
**SLC25A22**	-0.2194058	0.03049449	Yes
**UBAP2L**	0.07363605	0.6235542	Yes
**WDR59**	0.2273785	0.1046839	No
ACTR6	0.1109978	0.4389578	No
AES	-0.02050432	0.898471	Yes
AHCTF1	0.3730726	0.004460191	Yes
AP3D1	-0.2383877	0.08269878	No
API5	-0.1081276	0.4565956	No
ARHGEF2	-0.009367307	0.9545875	Yes
ARHGEF7	0.173763	0.1845487	No

ASCC1	0.4998325	0.002954352	Yes
ATN1	0.1217302	0.1737577	Yes
ATP5H	0.07525412	0.6188122	Yes
BASP1	-0.176706	0.2633279	Yes
BCAR3	-0.05367788	0.7422675	Yes
BCKDHB	-0.1942593	0.09301026	Yes
BRMS1L	0.1039899	0.4509514	Yes
BSDC1	0.09092808	0.551337	No
BST2	-0.490934	0.06802809	No
CA2	-0.06422838	0.2791562	Yes
CAPG	-0.6143357	0.000103104	No
CASC3	0.1625195	0.1721365	Yes
CDO1	1.829144	3.19E-07	Yes
CENPA	-0.08207925	0.4387936	No
CEP164	0.2432321	0.01370791	Yes
CERK	0.1343028	0.4278494	Yes
CH25H	-1.866276	1.45E-10	Yes
CHD9	0.1257607	0.3960325	Yes
CHFR	-0.2125413	0.1557228	Yes
CHPF	-0.1314065	0.3735946	Yes
CHST4	0.01558844	0.5488532	Yes
CIAPIN1	-0.07150332	0.5730652	Yes
CLPTM1	0.2432343	0.1278978	No
CLYBL	0.008894603	0.754284	No
CNOT3	0.0546751	0.4603973	Yes
CNTNAP1	-1.238708	6.13E-08	Yes
COQ3	-0.09855938	0.3535073	Yes
CPS1	0.2290268	0.1825634	Yes
DAAM1	-0.2430557	0.03356354	No
DDX18	0.1436745	0.3516404	Yes
DHPS	0.1440036	0.2489465	Yes

DHX16	0.3218946	0.02772507	Yes
DMWD	0.194658	0.1047469	Yes
DNAJB11	-0.5242841	0.01551282	No
DNASE1L3	-0.01143487	0.6727044	No
DPAGT1	0.04309813	0.7684001	Yes
DPP8	-0.06523132	0.6316888	No
DTX3L	0.01904233	0.8253489	Yes
DUT	0.1023102	0.3912785	Yes
DVL3	-0.06689867	0.6618635	Yes
EFTUD2	0.1438144	0.2966546	Yes
EHBP1	-0.08577124	0.5814841	No
EHD4	-0.5460186	0.002778402	Yes
EPM2AIP1	-0.1359256	0.3321766	Yes
ERCC5	0.2922338	0.06818784	Yes
FAM107B	-1.156139	5.11E-07	No
FBXO31	-0.01968161	0.8898534	Yes
FBXO32	1.570103	1.16E-09	Yes
FCRL4	-0.002745311	0.9090735	No
FEM1C	-0.1228183	0.3769151	Yes
GAL3ST4	-0.2513723	0.09192143	Yes
GALNT11	-0.004808426	0.9765335	Yes
GMDS	-0.5953401	0.001554946	Yes
GMIP	-0.1988042	0.03464187	No
GPAM	0.06356907	0.5585172	Yes
GPR63	-0.01718044	0.5493149	No
GPSM3	-0.02879238	0.4124842	No
GRWD1	-0.02176666	0.8649613	Yes
HDHD3	0.05072435	0.5058554	No
HECW2	-1.336751	3.99E-09	Yes
HMOX1	-0.4796912	0.02672771	Yes
HSD17B4	-0.06758181	0.6420411	Yes

HSD17B8	0.1427008	0.1871824	Yes
IFITM1	-0.4755904	0.08607455	No
IL12RB2	0.002337138	0.9275426	Yes
ILF3	-0.3373229	0.01956755	Yes
IMMP2L	0.4759356	0.007211556	Yes
IMP4	0.2827311	0.06226788	Yes
JAM3	-0.27854	0.07875547	No
LCMT2	-0.2105214	0.06002147	Yes
LMCD1	-0.05090523	0.8205146	Yes
LRG1	-0.0712328	0.1019569	No
LRMP	-0.05461915	0.1096815	No
LSS	0.07077058	0.365493	No
LTBR	0.4600118	0.003588852	Yes
LTV1	0.03768826	0.7911137	Yes
LYSMD2	-0.2976713	0.04295053	No
MAP3K11	-0.01176198	0.8706559	Yes
MAP3K9	0.03044987	0.3342286	Yes
MBTPS1	-0.05638059	0.6717802	No
MCCC1	1.095814	9.51E-07	Yes
MGRN1	-0.3428809	0.01200801	Yes
MIF4GD	0.09404469	0.4455904	Yes
MKNK1	0.1592128	0.2525494	Yes
MLKL	0.2149509	0.1116549	Yes
MNT	0.3214067	0.02800837	Yes
MRPL13	-0.382671	0.02169304	Yes
MRPL21	0.1453495	0.3361507	Yes
MYO9B	-0.2780479	0.01883349	No
NANOS1	-0.00412782	0.9115378	Yes
NDUFAF1	0.1486753	0.2690561	Yes
NETO2	-1.414598	2.99E-07	No
NFE2L1	0.3434404	0.02369207	Yes

NRIP1	0.2002312	0.1868392	Yes
OGFRL1	-0.01813634	0.71452	No
OPA1	0.01489544	0.8911344	Yes
OSBPL7	-0.1845357	0.06651421	Yes
PA2G4	0.1778768	0.1859495	Yes
PAK1IP1	-0.2853597	0.04565006	No
PAN3	0.1796872	0.1842327	Yes
PARP10	-0.257122	0.03600866	Yes
PDCD11	0.07384745	0.6243775	Yes
PDPR	0.1655366	0.2052807	Yes
PHF21A	0.05324777	0.7462746	Yes
PITPNM1	-0.04207103	0.7754115	Yes
PLCL2	0.000405947	0.9969239	Yes
PMM1	0.2566175	0.08698935	Yes
POLM	0.1265262	0.1672821	Yes
POLR1B	0.1015813	0.03515111	Yes
POMC	-0.06832727	0.1778132	Yes
POP1	-0.1296368	0.02369774	Yes
PPAT	0.02648512	0.8125248	Yes
PPP1R10	0.05345218	0.5740182	No
PRPF4	-0.01731237	0.8968255	Yes
PSMD1	-0.4296974	0.009754371	Yes
PSME3	-0.2313385	0.1277027	Yes
PSRC1	0.2598794	0.0647151	Yes
PTDSS2	0.04938412	0.6610378	Yes
PURB	0.1180083	0.45575	Yes
PYGO2	0.00981776	0.914171	Yes
RAC2	-0.6051047	0.003722524	Yes
RAF1	0.8533151	1.33E-06	Yes
RASL11A	1.193431	5.19E-11	Yes
RAVER1	-0.1529538	0.2343628	Yes

RFNG	-0.1386487	0.2927876	No
RNF10	-0.1178786	0.2626089	Yes
RPS29	0.02998606	0.6658347	Yes
SEMA4D	-0.01229191	0.7049854	Yes
SF3A1	0.335535	0.001977599	Yes
SH3BP1	-0.08612601	0.006560336	No
SLC16A9	-0.3364131	1.73E-05	Yes
SLC19A1	0.0235068	0.5599273	Yes
SLC1A1	-0.2268915	0.05093345	No
SLC39A14	0.2253844	0.1696513	No
SLC7A6	0.992774	5.26E-07	Yes
SLC8A3	0.04694271	0.1385412	No
SMG7	0.02756055	0.2155805	Yes
SMPD1	-0.3247134	0.06939667	Yes
SMYD2	0.04870574	0.7333661	Yes
SNCA	0.03009542	0.2019071	Yes
SNRPF	-0.1208207	0.4824207	Yes
SOD2	0.9655425	7.39E-06	Yes
SPTAN1	-0.09118875	0.5709116	Yes
SRRM1	-0.1024942	0.4354799	Yes
STAG3	0.02768771	0.3880839	No
STXBP2	-0.09626516	0.003201894	No
SUSD3	-0.03923035	0.1108457	No
SWAP70	0.6496999	0.000412843	Yes
SYK	-0.005101204	0.8340204	Yes
TBC1D17	0.1752784	0.05667533	Yes
TBK1	0.04910851	0.7368423	Yes
TCFL5	-0.1226683	0.3817121	Yes
TIFA	-0.003374418	0.9328039	No
TMCC3	0.03613917	0.1714005	No
TP53INP1	-0.003103256	0.9855975	Yes

TRAPPC1	-0.2654978	0.07782754	No
TRIB1	0.1084032	0.5323953	Yes
TRIM33	0.1115901	0.4216781	Yes
TTYH3	-0.8561338	1.19E-07	No
UBE2E1	0.2748404	0.08355346	Yes
UPF2	0.4067564	0.005752861	Yes
VHL	0.1119677	0.4338542	Yes
VPS37B	-0.03042062	0.7952246	Yes
WDR36	-0.1196588	0.3353938	Yes
WDR41	-0.3937136	0.021583	No
XAB2	0.4018402	0.008209929	Yes
YEATS2	-0.02515252	0.8630598	Yes
ZBED4	-0.1685489	0.07611387	Yes
ZBTB9	-0.1087732	0.2894304	Yes
ZCCHC11	0.6636235	0.000217712	Yes
ZHX1	-0.4054209	0.01785924	Yes
ZNF143	-0.02376938	0.8362303	Yes
ZNF451	-0.03834851	0.16816	Yes
ZNF622	0.762305	0.000111203	Yes

Bold denotes hub genes, [†]Results from GSE21727, [‡]Enriched pathway identified in this study

Table S3. Preservation statistics and P values of modules identified in gene expression profiles of Sham-treated immortalized B cells in gene expression profiles of Dex-treated immortalized B cells from childhood asthmatics

	Module	avg.weight	coherence	cor.cor	cor.degree	cor.contrib	avg.cor	avg.contrib
**Statistics**	Turquoise	0.04480772	0.3939333	0.9084948	0.9362422	0.9700504	0.3305905	0.569255
	Blue	0.01745769	0.2799837	0.8738084	0.9297415	0.9748477	0.2421161	0.4773045
	Brown	0.0190389	0.2947596	0.8656649	0.8930164	0.9594337	0.2497806	0.4839285
	Yellow	0.03345755	0.384043	0.8976904	0.8185916	0.9632477	0.3312046	0.5726935
	Green	0.03303415	0.3537517	0.869299	0.8185485	0.9455638	0.3061676	0.5390105
	Red	0.0209518	0.3138139	0.8614475	0.779286	0.9602717	0.2765148	0.5203651
	Black	0.02336877	0.327688	0.9012397	0.8693276	0.9762253	0.2929021	0.5329678
	Pink	0.02503431	0.3321562	0.8715029	0.8392627	0.9657686	0.2952158	0.5396827
	Magenta	0.01338425	0.2549678	0.8052235	0.6810665	0.9514463	0.2283777	0.4711403
	Purple	0.07344489	0.437857	0.8687456	0.9264696	0.9230179	0.3536952	0.5899504

	Turquoise	0.00009999	0.00009999	0.00009999	0.00009999	0.00009999	0.00009999	0.00009999
	Blue	0.00009999	0.00009999	0.00009999	0.00009999	0.00009999	0.00009999	0.00009999
	Brown	0.00009999	0.00009999	0.00009999	0.00009999	0.00009999	0.00009999	0.00009999
	Yellow	0.00009999	0.00009999	0.00009999	0.00009999	0.00009999	0.00009999	0.00009999
	Green	0.00009999	0.00009999	0.00009999	0.00009999	0.00009999	0.00009999	0.00009999
**P values**	Red	0.00009999	0.00009999	0.00009999	0.00009999	0.00009999	0.00009999	0.00009999
	Black	0.00009999	0.00009999	0.00009999	0.00009999	0.00009999	0.00009999	0.00009999
	Pink	0.00009999	0.00009999	0.00009999	0.00009999	0.00009999	0.00009999	0.00009999
	Magenta	0.09609039	0.00029997	0.00009999	0.00009999	0.00009999	0.00009999	0.00009999
	Purple	0.00009999	0.00009999	0.00009999	0.00009999	0.00009999	0.00009999	0.00009999

Table S4. The top-200 SNPs obtained from previous GWAS study

SNP	P value	SNP	P value	SNP	P value	SNP	P value	SNP	P value
rs7003550	1.46E-08	rs236113	3.43E-05	rs2346008	8.66E-05	rs6678151	0.0001397	rs6493265	0.0001891
rs9896933	3.15E-08	rs869697	3.66E-05	rs1369351	8.76E-05	rs303006	0.0001414	rs16940375	0.0001891
rs7599706	3.98E-08	rs11149155	3.90E-05	rs2273866	8.85E-05	rs6572555	0.0001443	rs12520537	0.0001896
rs12447718	4.26E-08	rs1209633	4.02E-05	rs17040590	8.88E-05	rs10262486	0.0001445	rs17015512	0.0001901
rs4368243	6.35E-08	rs1376478	4.11E-05	rs9831609	8.90E-05	rs303000	0.0001472	rs2825581	0.0001906
rs2316527	7.63E-08	rs4837698	4.13E-05	rs2824280	9.37E-05	rs4143188	0.0001483	rs1435218	0.0001909
rs4484658	8.09E-08	rs2840945	4.23E-05	rs7212240	9.68E-05	rs4480073	0.0001502	rs7689090	0.0001921
rs7506840	8.95E-08	rs4945688	4.36E-05	rs1208209	9.74E-05	rs6500586	0.0001529	rs715693	0.0001924
rs10485681	9.91E-08	rs10926977	4.44E-05	rs6428587	9.86E-05	rs12880735	0.0001535	rs4420519	0.0001928
rs3756612	1.94E-06	rs226313	4.53E-05	rs2825560	9.93E-05	rs11700399	0.0001544	rs2281169	0.0001955
rs12149765	2.09E-06	rs10511185	4.54E-05	rs939533	9.95E-05	rs1927731	0.0001556	rs17153785	0.0001963
rs10251582	4.58E-06	rs2048920	4.68E-05	rs12101676	9.95E-05	rs7403455	0.0001556	rs889104	0.0001969
rs2275732	4.67E-06	rs9341793	4.70E-05	rs4957696	9.97E-05	rs6951422	0.0001562	rs3745788	0.0001972
rs7818862	5.30E-06	rs4913287	4.79E-05	rs7587023	0.000101	rs1550948	0.0001573	rs9572552	0.0001976
rs12287409	6.13E-06	rs2572283	4.98E-05	rs10841528	0.0001016	rs220612	0.000159	rs12539392	0.0001989
rs7930174	6.13E-06	rs7139901	5.16E-05	rs8111710	0.0001022	rs1494023	0.00016	rs2099613	0.0001996
rs962963	7.93E-06	rs1945503	5.16E-05	rs17608067	0.0001023	rs242412	0.0001601	rs242405	0.0002001

rs2187331	8.03E-06	rs12118850	5.30E-05	rs6758317	0.0001025	rs136070	0.0001605	rs6930328	0.0002002
rs9473350	1.04E-05	rs764251	5.46E-05	rs6591350	0.0001031	rs17014050	0.0001645	rs763853	0.0002003
rs1911254	1.37E-05	rs9647406	5.52E-05	rs1782322	0.0001047	rs2030737	0.0001671	rs17094058	0.0002026
rs7822757	1.38E-05	rs12232191	5.65E-05	rs10801804	0.0001062	rs10087773	0.0001676	rs1956108	0.0002039
rs6602747	1.47E-05	rs4786860	5.80E-05	rs4495950	0.0001063	rs386790	0.0001683	rs6772123	0.00021
rs739994	1.70E-05	rs966935	5.83E-05	rs10464035	0.0001071	rs10243420	0.0001686	rs12918809	0.00021
rs770236	1.78E-05	rs1028458	6.15E-05	rs17217760	0.0001082	rs242401	0.0001691	rs1469572	0.0002112
rs4853475	1.81E-05	rs2386983	6.21E-05	rs1040151	0.0001089	rs12740041	0.00017	rs281505	0.0002129
rs1367685	1.82E-05	rs6845304	6.27E-05	rs675566	0.0001103	rs6023633	0.0001707	rs1462831	0.0002134
rs1896950	1.90E-05	rs10514688	6.28E-05	rs2182954	0.0001112	rs6957858	0.0001714	rs2146880	0.0002137
rs4886620	2.11E-05	rs9915334	6.43E-05	rs6963373	0.0001144	rs4864471	0.0001741	rs2436634	0.0002166
rs1903876	2.15E-05	rs7176566	6.48E-05	rs1126328	0.0001168	rs12639326	0.0001746	rs2814848	0.0002173
rs8074277	2.20E-05	rs6771233	6.52E-05	rs220611	0.0001196	rs7835456	0.0001748	rs1996709	0.0002176
rs2117906	2.21E-05	rs2824317	7.01E-05	rs10778889	0.0001198	rs10494229	0.0001775	rs2073831	0.0002178
rs3743487	2.52E-05	rs1239104	7.07E-05	rs10791993	0.0001211	rs6130139	0.0001785	rs7166898	0.0002178
rs7149088	2.65E-05	rs2401035	7.19E-05	rs2001558	0.0001217	rs11252485	0.0001794	rs2825544	0.0002179
rs998235	2.76E-05	rs1394551	7.41E-05	rs3810524	0.0001245	rs9856875	0.0001795	rs2347935	0.0002196
rs11058317	2.78E-05	rs10257931	7.61E-05	rs2341459	0.0001263	rs11242598	0.0001799	rs7097254	0.0002199
rs1011692	2.91E-05	rs12432642	7.69E-05	rs1105274	0.0001266	rs1949929	0.0001805	rs1438182	0.0002223
rs7154679	2.99E-05	rs9817986	7.78E-05	rs1385432	0.0001275	rs2732231	0.0001816	rs2930796	0.0002225

rs17678758	3.18E-05	rs4779618	7.92E-05	rs1468627	0.0001284	rs9888659	0.0001818	rs2144800	0.0002225
rs4677414	3.34E-05	rs4877971	8.11E-05	rs10801805	0.000133	rs2007888	0.0001843	rs16983718	0.0002228
rs236111	3.43E-05	rs10812520	8.37E-05	rs12232511	0.0001348	rs4336567	0.0001884	rs12649285	0.0002265

Table S5. Preservation statistics and P values of modules identified in gene expression profiles of Sham-treated immortalized B cells in gene expression profiles of Sham-treated osteoblast from GSE21727

	**Module**	**avg.weight**	**coherence**	**cor.cor**	**cor.degree**	**cor.contrib**	**avg.cor**	**avg.contrib**
**Statistics**	Turquoise	0.6544096	0.6031521	0.04222102	0.117234467	0.12782118	0.012455046	0.08988802
	Blue	0.6514467	0.5967116	0.03307033	0.046351508	0.11102283	0.019290233	0.11550161
	Brown	0.6537856	0.603804	0.03447525	0.00710907	0.06099031	0.017651138	0.06263249
	Yellow	0.6799488	0.6645311	0.46249519	0.300256852	0.73507939	0.319278805	0.57574776
	Green	0.6822337	0.6677667	0.3033763	0.253927274	0.49400758	0.249250918	0.50551534
	Red	0.6781878	0.6600332	0.32291358	0.086556734	-0.62991583	0.212733097	-0.45032814
	Black	0.6460818	0.5811741	0.01554291	0.071296112	0.04287394	0.008792729	0.0678776
	Pink	0.6525093	0.6015149	0.04848967	0.007140164	0.19079314	0.023572609	0.1516882
	Magenta	0.6917683	0.6869658	0.29738041	0.326949677	0.47228733	0.238804094	0.4903798
	Purple	0.7006673	0.7004956	0.28914427	0.264062108	0.41262065	0.350409141	0.59139682

**P values**	Turquoise	0.6520348	0.70482952	0.00009999	0.00009999	0.00009999	0.00009999	0.00089991
	Blue	0.84531547	0.83811619	0.00009999	0.09829017	0.0009999	0.00009999	0.00049995
	Brown	0.63973603	0.63073693	0.00009999	0.4279572	0.06079392	0.00009999	0.02569743
	Yellow	0.00029997	0.00029997	0.00009999	0.00009999	0.00009999	0.00009999	0.00009999
	Green	0.00039996	0.00029997	0.00009999	0.00009999	0.00009999	0.00009999	0.00009999
	Red	0.00089991	0.00119988	0.00009999	0.0489951	1	0.00009999	1
	Black	0.93080692	0.92890711	0.01449855	0.10508949	0.2279772	0.02959704	0.10238976
	Pink	0.61383862	0.64013599	0.00059994	0.46605339	0.0019998	0.00449955	0.00559944
	Magenta	0.00059994	0.00039996	0.00009999	0.00009999	0.00009999	0.00009999	0.00009999
	Purple	0.00209979	0.00179982	0.00009999	0.00139986	0.00009999	0.00009999	0.00009999

Table S6. Hub genes of the blue module (childhood asthmatics-specific gene module)

Gene	Gene	Gene	Gene	Gene
ZDHHC23	RSAD1	CTNNBIP1	TRAK2	SGPP1
TRRAP	PTAFR	SMARCD1	TRIP13	ZNF337
TOB2	POLR2B	RBM15	UBQLN4	CHKA
CELSR3	BLM	CUL1	NT5E	SNRK
LRFN4	PISD	PAQR3	SUPT16H	CCT5
DENND1A	ACAD10	GART	CBFB	SBF1
RAB6A	PIK3R4	RICTOR	KIAA0922	DLL3
RPS6KA5	RNPEPL1	CYLD	MTAP	TTLL12
CSE1L	TRAF5	GPD1L	ABCA3	JSRP1
STAT5B	FBXO21	MYL9	CEBPG	MYO1D
TFAP4	PHF10	TATDN2	ETV6	RNH1
PAK2	BICD2	DCBLD2	ZNF562	
UBE3B	MESDC1	ATP1A1	EXOC6	
TRIM47	RASGRP2	MCM3AP	MCM6	
EML4	TMTC4	RPS6KB1	PSME4	
LMNB2	ETV4	ZNF281	S100A10	
GANAB	ACY3	PRCC	ACOT4	

Table S7. Biological pathways enriched in the blue module (childhood asthmatics-specific gene module)

ID	Name[†]	Depth[‡]	P value[§]
GO:0051348	Negative regulation of transferase activity	1	0.0112
GO:0071705	Nitrogen compound transport	1	0.00577
GO:0033036	Macromolecule localization	1	0.000083
GO:0008104	Protein localization	2	0.0000187
GO:0045184	Establishment of protein localization	3	0.00211
GO:0051641	Cellular localization	1	0.000000237
GO:0070727	Cellular macromolecule localization	2	0.000166
GO:0034613	Cellular protein localization	3	0.000215
GO:0051649	Establishment of localization in cell	1	0.0000789
GO:0046907	Intracellular transport	2	0.000269
GO:0035556	Intracellular signal transduction	1	0.0387
GO:0007249	I-kappaB kinase/NF-kappaB signaling	2	0.00979
GO:0043122	Regulation of I-kappaB kinase/NF-kappaB signaling	1	0.00348
GO:0008150	Biological_process	1	5.55E-08
GO:0071840	Cellular component organization or biogenesis	2	0.00000651
GO:0044085	Cellular component biogenesis	3	0.00512
GO:0002376	Immune system process	2	0.000014

GO:0044699	Single-organism process	2	0.00000172
GO:0008152	Metabolic process	2	1.17E-08
GO:0044238	Primary metabolic process	3	0.000000726
GO:0009058	Biosynthetic process	3	0.0151
GO:0009056	Catabolic process	3	0.00000161
GO:0071704	Organic substance metabolic process	3	0.000000274
GO:0006807	Nitrogen compound metabolic process	3	0.0000099
GO:0050896	Response to stimulus	2	0.0025
GO:0042221	Response to chemical	3	0.00178
GO:0009628	Response to abiotic stimulus	3	0.00735
GO:0006950	Response to stress	3	0.00000223
GO:0009607	Response to biotic stimulus	3	0.0176
GO:0006955	Immune response	3	0.00866
GO:0065007	Biological regulation	2	0.000416
GO:0065009	Regulation of molecular function	3	0.00285
GO:0051704	Multi-organism process	2	0.011
GO:0044419	Interspecies interaction between organisms	3	0.0001
GO:0051707	Response to other organism	3	0.00675
GO:0009987	Cellular process	2	1.15E-10
GO:0044763	Single-organism cellular process	3	0.00000227

GO:0016043	Cellular component organization	3	0.0000855
GO:0044237	Cellular metabolic process	3	7.89E-11
GO:0051716	Cellular response to stimulus	3	0.00772
GO:0022402	Cell cycle process	3	0.0000554
GO:0050789	Regulation of biological process	2	0.000326
GO:0050794	Regulation of cellular process	3	0.00472
GO:0019222	Regulation of metabolic process	3	0.000388
GO:0048519	Negative regulation of biological process	2	0.00000102
GO:0048523	Negative regulation of cellular process	3	0.0000874
GO:0009892	Negative regulation of metabolic process	3	0.0114
GO:0048518	Positive regulation of biological process	2	0.00034
GO:0048522	Positive regulation of cellular process	3	0.0372
GO:0002684	Positive regulation of immune system process	3	0.00615
GO:1903320	Regulation of protein modification by small protein conjugation or removal	1	0.000011
GO:1903322	Positive regulation of protein modification by small protein conjugation or removal	2	0.0183
GO:0031396	Regulation of protein ubiquitination	2	0.0000167
GO:0031398	Positive regulation of protein ubiquitination	3	0.0499
GO:0031400	Negative regulation of protein modification process	1	0.0136
GO:1903321	Negative regulation of protein modification by small protein conjugation or removal	2	0.00468
GO:0031397	Negative regulation of protein ubiquitination	3	0.0016

GO:0031349	Positive regulation of defense response	1	0.005
GO:0045089	Positive regulation of innate immune response	2	0.000201
GO:0002218	Activation of innate immune response	3	0.000223
GO:0010243	Response to organonitrogen compound	1	0.0346
GO:0070646	Protein modification by small protein removal	1	0.0202
GO:0016579	Protein deubiquitination	2	0.00678
GO:0051603	Proteolysis involved in cellular protein catabolic process	1	0.00416
GO:0019941	Modification-dependent protein catabolic process	2	0.0045
GO:0006511	Ubiquitin-dependent protein catabolic process	3	0.00729
GO:0002758	Innate immune response-activating signal transduction	1	0.000121
GO:0007159	Leukocyte cell-cell adhesion	1	0.000436
GO:1903037	Regulation of leukocyte cell-cell adhesion	2	0.00417
GO:0050863	Regulation of T cell activation	1	0.00408
GO:2001235	Positive regulation of apoptotic signaling pathway	1	0.0458

[†]Gene ontology biological pathway, [‡]Only depth 1 ~ 3 were presented, [§]Benjamini-Hocherg FDR P value
